# Altered structural and functional connectivity between the bilateral primary motor cortex in unilateral subcortical stroke: A multimodal magnetic resonance imaging study

**DOI:** 10.1097/MD.0000000000004534

**Published:** 2016-08-07

**Authors:** Yong Zhang, Kuang-Shi Li, Yan-Zhe Ning, Cai-Hong Fu, Hong-Wei Liu, Xiao Han, Fang-Yuan Cui, Yi Ren, Yi-Huai Zou

**Affiliations:** a Department of Neurology and Stroke Center, Dongzhimen Hospital, The First Affiliated Hospital of Beijing University of Chinese Medicine; b Department of Emergency, Beijing Gulou Hospital of Traditional Chinese Medicine, Beijing, China.

**Keywords:** functional magnetic resonance imaging, primary motor cortex, stroke

## Abstract

A large number of functional imaging studies have focused on the understanding of motor-related neural activities after ischemic stroke. However, the knowledge is still limited in the structural and functional changes of the interhemispheric connections of the bilateral primary motor cortices (M1s) and their potential influence on motor function recovery following stroke.

Twenty-four stroke patients with right hemispheric subcortical infarcts and 25 control subjects were recruited to undergo multimodal magnetic resonance imaging examinations. Structural impairments between the bilateral M1s were measured by fractional anisotropy. Functional changes of the bilateral M1s were assessed via M1-M1 resting-state functional connectivity. Task-evoked activation analysis was applied to identify the roles of the bilateral hemispheres in motor function recovery. Compared with control subjects, unilateral subcortical stroke patients revealed significantly decreased fractional anisotropy and functional connectivity between the bilateral M1s. Stroke patients also revealed higher activations in multiple brain regions in both hemispheres and that more regions were located in the contralesional hemisphere.

This study increased our understanding of the structural and functional alterations between the bilateral M1s that occur in unilateral subcortical stroke and provided further evidence for the compensatory role played by the contralesional hemisphere for these alterations during motor function recovery.

## 1 Introduction

Stroke has become the leading cause of motor disability worldwide among adults.^[1]^ It results from the loss of blood supply in certain brain tissues which will eventually lead to motor impairment. A large number of functional imaging studies have focused on the understanding of motor-related neural activities after ischemic stroke.^[2]^ The primary motor cortex (M1) is the principal brain region of the motor circuit that involves in motor recovery.^[3]^ Its major function is to plan and execute contralateral voluntary movements with other motor and subcortical regions. It has been demonstrated by previous studies that structural and functional alterations exist in the ipsilesional and contralesional M1s in stroke patients.^[4,5]^ However, the association between the structural and functional changes of the interhemispheric connections of the bilateral M1s and their potential influence on motor recovery after stroke remains largely unknown.^[6]^ Hence, systematic investigation into the altered connectivity between the bilateral M1s is still a matter of active in stroke research and will provide further understanding for the mechanisms of motor recovery.

Structural and functional changes following stroke have been extensively investigated with various functional magnetic resonance imaging (fMRI) techniques. Diffusion tensor imaging (DTI) is used to quantitatively measure the structural connectivity of white matter tracts and provide information of cellular integrity.^[7–9]^ Decreased fractional anisotropy (FA) has been consistently proven in the affected corticospinal tract (CST) across studies in subcortical stroke involving motor pathways.^[10,11]^ The resting-state functional connectivity (FC) is another widely used technique to evaluate the temporal correlations between any pair of predefined brain regions.^[12–14]^ It has been demonstrated by previous studies that most stroke patients exhibit reduced FC between the bilateral M1s compared with normal subjects.^[5,15,16]^ Task-evoked activation analysis has also been extensively applied to study the underlying mechanisms of motor function reorganization during stroke recovery.^[17–19]^ There is a high consistency that movements of the stroke affected hand are associated with increased activations in the bilateral M1s.^[20,21]^

In the current study, we conducted a multimodal MRI study by combining DTI, resting-sate FC, and task-evoked activation analysis simultaneously to examine the changes of interhemispheric connections of the bilateral M1s during motor recovery in stroke patients. We hypothesized that interhemispheric functional and structural damages between the bilateral M1s occur following subcortical stroke and that enhanced functional reorganization of the bilateral hemispheres during hand movement task may exist to compensate for these damages. Structural impairments between the bilateral M1s were measured by the FA of M1-M1 anatomic connection. Functional changes of the bilateral M1s were assessed via M1-M1 resting-state FC. We also applied task-evoked activation analysis to identify the roles of the bilateral hemispheres in motor function reorganization. In this study, only ischemic stroke patients with subcortical infarctions involving the motor pathways were included. In order to eliminate the dominant effect of the left hemisphere, we only recruited patients with right hemispheric lesions who were right-handed before stroke.

## 2 Materials and methods

### 2.1 Subjects

**Figure 1:**
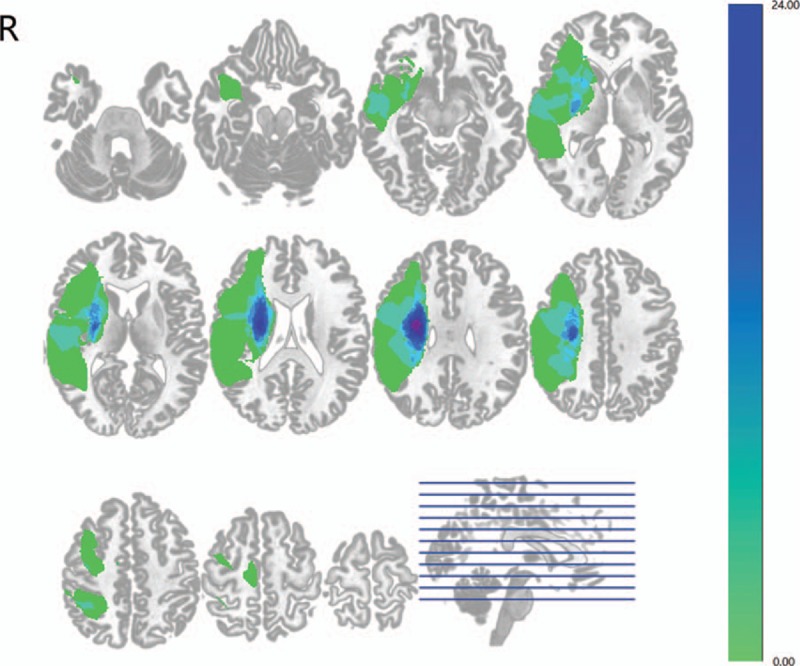
Lesion incidence map of right hemispheric subcortical stroke patients. Patients with subcortical infarctions involving the right motor pathways were included in the current study.

A total of 24 patients with right hemispheric subcortical infarcts were recruited (17 male, aged 62.83 ± 8.44 years). The basic demographic and clinical features are shown in Table 1. The lesion incidence map of all patients is shown in Fig. 1. The inclusion criteria were as follows: first onset of ischemic stroke; aged 35 to 75 years; single subcortical lesion restricted to the right internal capsule, basal ganglia, corona radiate, and its neighboring regions; right handed before stroke; 2 weeks to 6 months after the onset of stroke; without neurologic or psychiatric disorders. The exclusion criteria were as follows: recurrent stroke; any brain abnormalities; any history of alcohol or drug dependency; any other health problems or poor physical conditions that may influence participation; any MRI contraindications. Another 25 healthy subjects (13 male, aged 59.50 ± 6.31 years) with no history of neurologic or psychiatric disorders were recruited as the control group. The study was approved by the Research Ethical Committee of Dongzhimen Hospital affiliated to Beijing University of Chinese Medicine and conducted in accordance with the Declaration of Helsinki. Written informed consents were obtained from all participants.

**Table 1:**
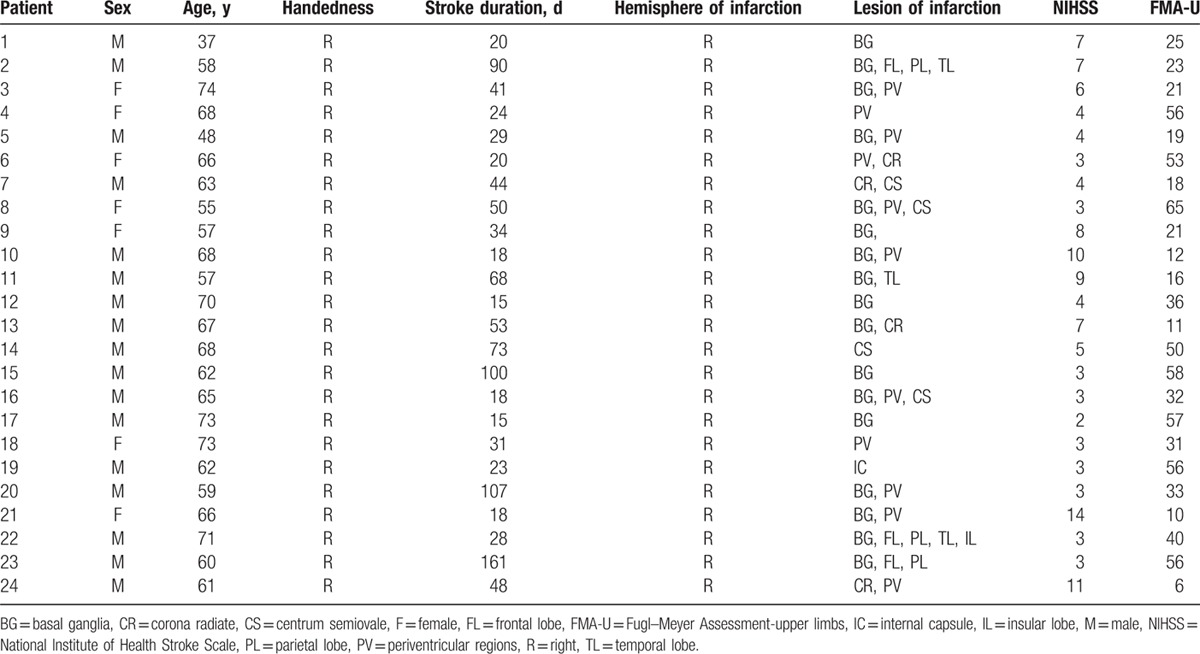
The basic demographic and clinical data of right hemispheric subcortical stroke patients.

### 2.2 Imaging acquisition

Images were acquired using a 3.0 Tesla MRI scanner (Siemens, Sonata Germany) at Dongzhimen Hospital, Beijing, China. Prior to scanning, all participants were asked to rest for 20 minutes and were instructed to stay still, think of nothing in particular, keep eyes closed, and not to fall asleep during scanning. Earplugs were worn to attenuate scanner noise and foam head holders were immobilized to minimize head movements during scanning.

Prior to the functional scanning, high-resolution structural information for anatomical localization was acquired using 3D MRI sequences with the following parameters: voxel size = 1 mm^3^, repetition time = 1900 ms, echo time = 2.52 ms, flip angle = 90°, matrix = 256 × 256, field of view = 250 mm × 250 mm, slice thickness = 1 mm.

The DTI data were obtained with a single-shot, gradient-recalled echo-planar imaging sequence. The diffusion sensitizing gradients were applied along 30 noncollinear directions (*b* = 1000 s/mm^2^) with an acquisition without diffusion weighting (*b* = 0 s/mm^2^). The imaging parameters were as follows: repetition time = 18,000 ms, echo time = 94 ms, flip angle = 90°, matrix = 160 × 160, field of view = 256 mm × 256 mm, slice thickness = 1.5 mm, 80 contiguous axial slices.

Resting-state fMRI data was collected using a single-shot, gradient-recalled echo-planar imaging sequence with the following parameters: repetition time = 2000 ms, echo time = 30 ms, flip angle = 90°, matrix = 64 × 64, field of view = 225 mm × 225 mm, slice thickness = 3.5 mm, gap = 1 mm, 32 interleaved axial slices, and 180 volumes. The same parameters to resting-state scanning were used in the task-evoked fMRI with exception that 100 volumes were acquired.

### 2.3 Task design

All participants received a passive finger-grasping task during the task-evoked fMRI scanning. The task was performed on the left thumb and index finger by a trained researcher at a frequency of 1.0 Hz. The task cycle consisted of a 20-second testing block followed by a 20-second movement and was repeated for 5 times during the scanning. The reason why we chose passive movement was to avoid the limitations of participants’ mirror movement and motor expectation which usually appear in voluntary movement.

### 2.4 Data processing and multimodal fMRI analysis

The DTI data processing and analyzing were mainly carried out using the FMRIB Software Library (FSL) and Analysis of Functional NeuroImage (AFNI) software.^[22]^ The original data was first corrected for eddy distortion and head motion. The brain extraction toolbox was used for brain extraction. Subsequently, the extracted images of each subject were affinely coregistered into T1 template. Diffusion tensor was calculated with the AFNI software. Then, the DTITK software was applied to produce the final average template of all subjects. Tract-based spatial statistics were used for FA calculation. For the comparison between 2 groups, results were analyzed with the FSL randomized model test with the FEW multiple corrections.

The resting-state data processing and analyzing were manly carried out with the statistical parametric mapping software (SPM12). A total of 174 volumes for each subject were corrected for slice timing after the starting volumes were discarded for signal equilibrium. The following steps were spatial realignment for head motions, normalization into the Montreal Neurological Institute template, resampling into 3 × 3 × 3 mm^3^ voxels, smoothing with a Gaussian kernel of 6 × 6 × 6 mm^3^ full width at half-maximum, and linear regression. The FC of 2 groups was conducted by 2-sample *T* test. Similar processing steps were applied to the task-evoked data. For region of interest (ROI) level analysis, the bilateral precentral gyri were selected as ROIs with the AAL template. The mean time series of both ROIs were extracted and converted to z scores using Fisher r-to-z transformation to normality. Two-sample *T* test (2-tailed, *P* <0.05, Monte Carlo Simulations correction) was conducted between 2 groups. The reported statistics were color-coded and mapped in Talairach space.

## 3 Results

### 3.1 DTI results

**Figure 2:**
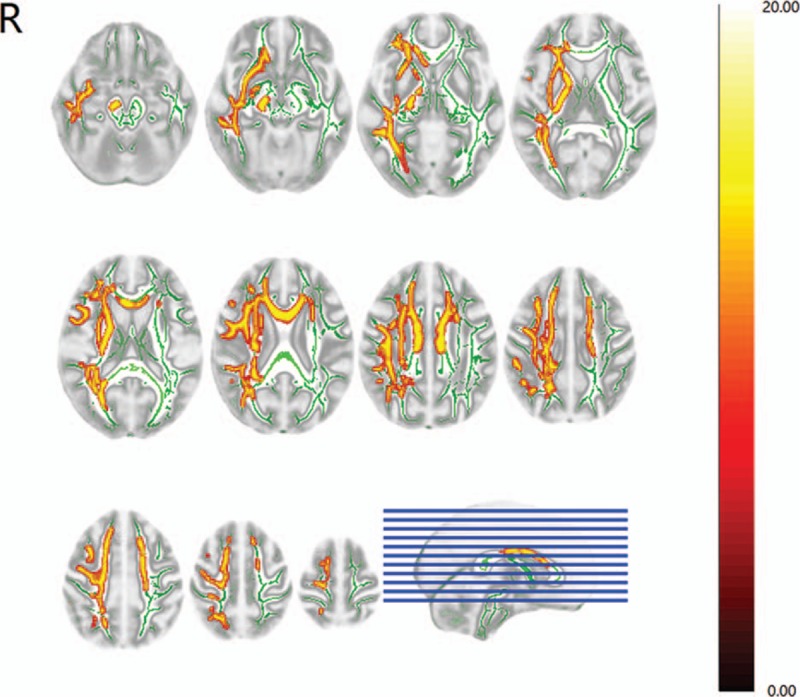
Diffusion tensor imaging results of the interhemispheric anatomic connection between the bilateral primary motor cortices and the bilateral corticospinal tracts. Right hemispheric subcortical stroke patients showed significantly decreased fractional anisotropy in the corpus callosum and right corticospinal tract.

The corpus callosum is the main interhemispheric structural interaction between the bilateral M1s. We compared the FA of the corpus callosum between stroke patients and control subjects to investigate the structural impairments of M1-M1 anatomic connection. Stroke patients revealed significantly decreased FA in the corpus callosum compared with control subjects (Fig. 2). We also performed comparison of the bilateral CSTs within and between 2 groups. The right CST in stroke patients, which was affected by subcortical infarctions, revealed significantly decreased FA compared with the left CST and the bilateral CSTs in control subjects (Fig. 2). There were no differences detected among stroke patients’ left CST and control subjects’ bilateral CSTs.

### 3.2 Resting-state FC results

Changes of the resting-state FC between the bilateral M1s were studies to assess the functional impairments following stroke. Compared with control subjects, unilateral subcortical stroke patients with infarctions in the motor pathway revealed decreased resting-state FC between the bilateral M1s (*P* = 0.006, *T* = 2.86).

### 3.3 Task activation results

**Figure 3:**
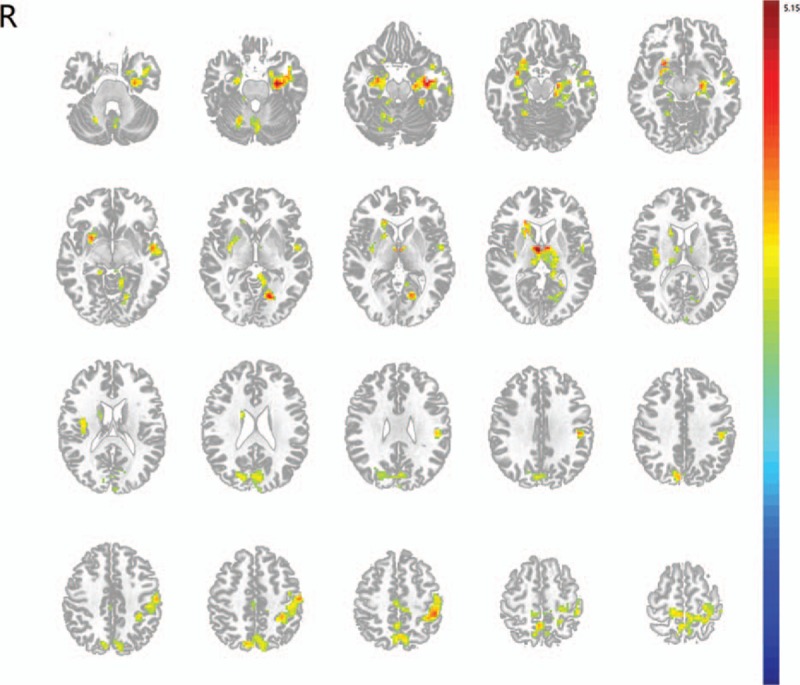
Motor task activation map. Right hemispheric subcortical stroke patients revealed significant higher activations in a series of brain regions following left hand motor task.

The motor task activated multiple motor related brain regions including the primary sensorimotor areas and the secondary motor areas of both hemispheres in stroke patients and control subjects. The right hemisphere affected subcortical stroke patients revealed significantly higher activations in a series of brain regions following left hand motor task. The regions that showed higher activations in stroke patients included the left postcentral gyrus, left precentral gyrus, left middle temporal gyrus, left inferior parietal lobule, bilateral paracentral lobule, bilateral thalamus, bilateral parahippocampal gyrus, bilateral precuneus, and bilateral cuneus (see Fig. 3 and Table 2). There were no regions that showed decreased activations in stroke patients compared with control subjects.

**Table 2:**
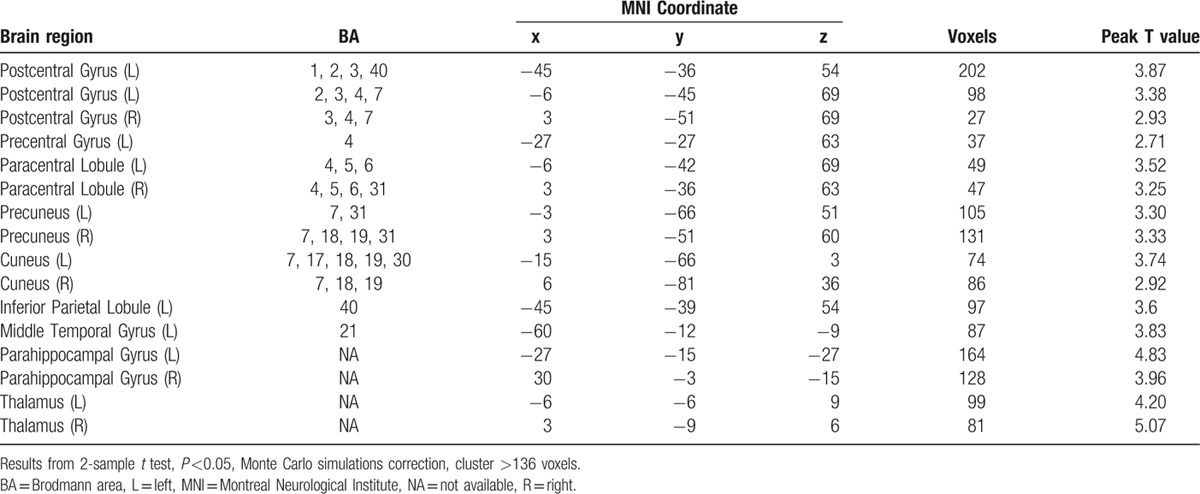
Brain regions that showed higher activations following left hand motor task in stroke patients compared to control subjects.

## 4 Discussion

In the current multimodal MRI study, we recruited right hemisphere damaged subcortical stroke patients and investigated the structural and functional changes between the bilateral M1s. Stroke patients exhibited decreased FA and FC between the bilateral M1s which indicated alerted structural and functional interhemispheric connections between the bilateral M1s. Moreover, a series of brain regions related to motor function revealed increased activations following left-hand movements in stroke patients which might serve as a compensation for the M1-M1structural and functional impairments.

Most previous studies investigating the structural and functional impairments of stroke involved patients with infarctions in both hemispheres. Given the presence of functional asymmetry between the right and left hemispheres during resting state, it is reasonable and necessary to eliminate the potential influence of lateralization of the dominant hemisphere in fMRI studies.^[23]^ The role of the right hemisphere in motor function related tasks has been highlighted in right-handed hemiplegic patients.^[24]^ In another recent fMRI study focusing on the structural and functional changes following stroke, right hemisphere damaged patients revealed more significant alterations in subgroup analysis.^[25]^ These results suggested that the right hemisphere (in right-handed individuals) may play a more important role in relation to motor functions in stroke patients. Thus, we proposed that the results from right hemisphere damaged patients would be more convincing and accurate in determining the structural and functional alterations following stroke.

We repeated the results of previous studies involving infarctions in both hemispheres that reported decreased FA in the corpus callosum indicating structural impairments of the M1-M1 connections in subcortical stroke patients.^[5,25–27]^ Meanwhile, decreased FA in the stroke affected right CST was also detected which was consistent with previous studies showing FA reductions within 6 months or an even longer time after the onset of stroke.^[10,11,28,29]^ Originating form M1, the CST serves as the most crucial outflow tract of the motor system. It has been demonstrated that alterations of its structural integrity would critically influence the motor function recovery in stroke patients.^[10,11,28,29]^ The impaired M1-M1 anatomic connections are believed to result from trans-synaptic axonal denegation and secondary to the structural integrity damages of the CST as both fibers are connected with neurons of M1.^[25]^ As the main interhemispheric structural interactions between the bilateral M1s, the corpus callosum plays an important role in modulating cortical motor functions and motor performances. Previous fMRI study has indicated that the damaged structural integrity of the corpus callosum is a relevant factor that influences motor function recovery in stroke patients.^[26]^ Determining the structural connectivity alterations between the bilateral M1s may provide pivotal insights into the functional role of stroke-related changes in the underlying structural networks.^[4]^

The M1-M1 resting-state FC is highly associated with motor function recovery in subcortical stroke patients.^[30]^ Many studies have investigated the resting-state FC between the bilateral M1s and revealed an extensive dynamic evolution during stroke recovery. The M1-M1 resting-state FC has been found to be significantly decreased in the initial stages of stroke and gradually increase to a level near normal after 6 months or even a longer time.^[5,31,32]^ In the current study, we only included patients within 6 months after the onset of stroke. We demonstrated decreased resting-state FC between the bilateral M1s which was highly consistent with previous findings. The reduced FC implies the breakdown of harmonious interaction between bilateral M1s after subcortical stroke.^[33]^ However, a recent fMRI study, which detected similar findings of M1-M1 structural alterations, revealed increased FC between the bilateral M1s in subcortical stroke patients.^[25]^ We speculated that the differences in stroke stages may account for this discrepancy. They only recruited patients at a stable chronic stage with good outcome of motor function recovery. We believe that the existing results are compatible as the decreased M1-M1 FC may reach a higher level in the dynamic evolution when patients gained good recovery.

We also performed task-evoked activation analysis to further investigate the possible underlying mechanisms of motor function recovery following stroke. Our results showed that motor task of the stroke affected hand initially hyperactivated extensive brain regions in both ipsilesional and contralesional hemispheres and that more regions were located in the contralesional hemisphere. Our findings coincided with precious task-related fMRI studies that reported increased activations in motor-related regions and nonmotor brain areas in stroke patients.^[6,31,32,34,35]^ Increased activations in the ipsilesional hemisphere indicated that the structural damaged regions retain the potential of functional reorganization during motor recovery.^[6]^ For the enhanced activities in the contralesional hemisphere, it has been suggested to impair the recovery of motor function in several studies using transcranial magnetic stimulation.^[36,37]^ However, results from a large number of longitudinal studies have provided converging evidence that enhanced activities in the contralesional hemisphere reflect the compensatory plastic changes of the brain function for the stroke-affected interhemispheric structural and functional connectivity. ^[5,6,31,32,34,35]^

By combining the results of DTI, resting-state FC, and task-evoked activation analysis, we have provided a broad view from different aspects into the structural and functional alterations between bilateral M1s and related compensatory plastic changes for motor recovery in stroke patients. It is of great clinical significance to determining the structural and functional alterations between the bilateral M1s and related beneficial roles in motor functional recovery in subcortical stroke patients. The interhemispheric structural and functional connectivity changes between the bilateral M1s may serve as neurobiological markers for the evaluation of motor function recovery. ^[27,38]^ They could provide valuable information for the prognosis and prediction of motor function recovery following stroke. Recent fMRI studies have revealed increased M1-M1 connectivity and positive relationship with motor function recovery after clinical treatment in subcortical stroke patients.^[5,27,38–40]^ Characterizing the alterations of M1-M1 connectivity in stroke survivors may help optimize rehabilitation therapies targeting specific motor pathways.^[41]^ Moreover, further investigation into how the bilateral hemispheres interact during motor function recovery will add better understanding of brain plasticity and reorganization during stroke recovery.^[42,43]^

The current study was restricted to several limitations. The links between the structural and functional alterations and the patients’ behavioral data were not included in the current study. Future studies should focus on the correlations of imaging results and clinical scales to provide further interpretations in this field. We recruited patients from 2 weeks to 6 months after the onset of stroke which is a wide range of stroke stage. Our results need to be further validated in more specified groups of patients and long-term longitudinal studies covering all stages of stroke. Studies recruiting a larger sample size with a larger range of stroke stage and more behavioral data are still needed to further confirm our results and to provide beneficial information for clinical rehabilitation during motor function recovery followings stroke.

## 5 Conclusions

In conclusion, this study increased our understanding of the structural and functional alterations between the bilateral M1s that occur in unilateral subcortical stroke and provided further evidence for the compensatory role played by the contralesional hemisphere for these alterations during motor function recovery.
